# Parametric Pattern Selection in a Reaction-Diffusion Model

**DOI:** 10.1371/journal.pone.0077337

**Published:** 2013-10-29

**Authors:** Michael Stich, Gourab Ghoshal, Juan Pérez-Mercader

**Affiliations:** 1 Department of Earth and Planetary Sciences, Harvard University, Cambridge, Massachusetts, United States of America; 2 The Santa Fe Institute, Santa Fe, New Mexico, United States of America; Universitat Politecnica de Catalunya, Spain

## Abstract

We compare spot patterns generated by Turing mechanisms with those generated by replication cascades, in a model one-dimensional reaction-diffusion system. We determine the stability region of spot solutions in parameter space as a function of a natural control parameter (feed-rate) where degenerate patterns with different numbers of spots coexist for a fixed feed-rate. While it is possible to generate identical patterns via both mechanisms, we show that replication cascades lead to a wider choice of pattern profiles that can be selected through a tuning of the feed-rate, exploiting hysteresis and directionality effects of the different pattern pathways.

## Introduction

Reaction-diffusion systems are well known to self-organize into a variety of spatio-temporal patterns including, spots, stripes, spirals, as well as spatio-temporal chaos and uniform oscillations [1]–[3]. Their existence in out-of-equilibrium states, connection to idealized chemical systems, and dependence on dimensional parameters, make them a good testbench for the study of general features of pattern generation and evolution. In particular, the dependence of these final states on the rate at which constituents are fed into the system (feed-rate) is of significant interest, since reaction-diffusion systems represent proxies for high-level biological systems that can exchange matter and energy with the environment [4]. Depending on the value of the feed-rate, the system may asymptote into one of many states and thus the feed-rate can be thought of playing the role of a natural control parameter.

While spatio-temporal patterns in reaction-diffusion systems (like replicating spots [5] and Turing patterns [6], [7]) have been found and discussed extensively in the context of chemical systems [3], [8], their phenomenology is ubiquitous. A well-studied example from physics is related to electrical current filament patterns in planar gas-discharge systems [9], [10]. The system dynamics can be described by activator-inhibitor reaction-diffusion models and different mechanisms of spot array formation have been observed: division and self-completion. The relevant control parameter in this system is the feeding voltage. Another example that have attracted interest recently is found in the realm of fluid dynamics where “spots” of turbulent regions in pipe flow [11] and plane Couette flow [12] have been observed: On a laminar background, patches of localized turbulence, called puffs, emerge via finite-amplitude perturbations and also show splitting behavior. These systems have been recently mapped onto excitable reaction-diffusion systems [13], and subsequently, the Turing mechanism has been proposed to explain the periodic arrangement of puffs in [14], suggesting again a reaction-diffusion framework for the dynamics. The corresponding control parameter in this case is the Reynolds number of the flow.

**Figure 1:**
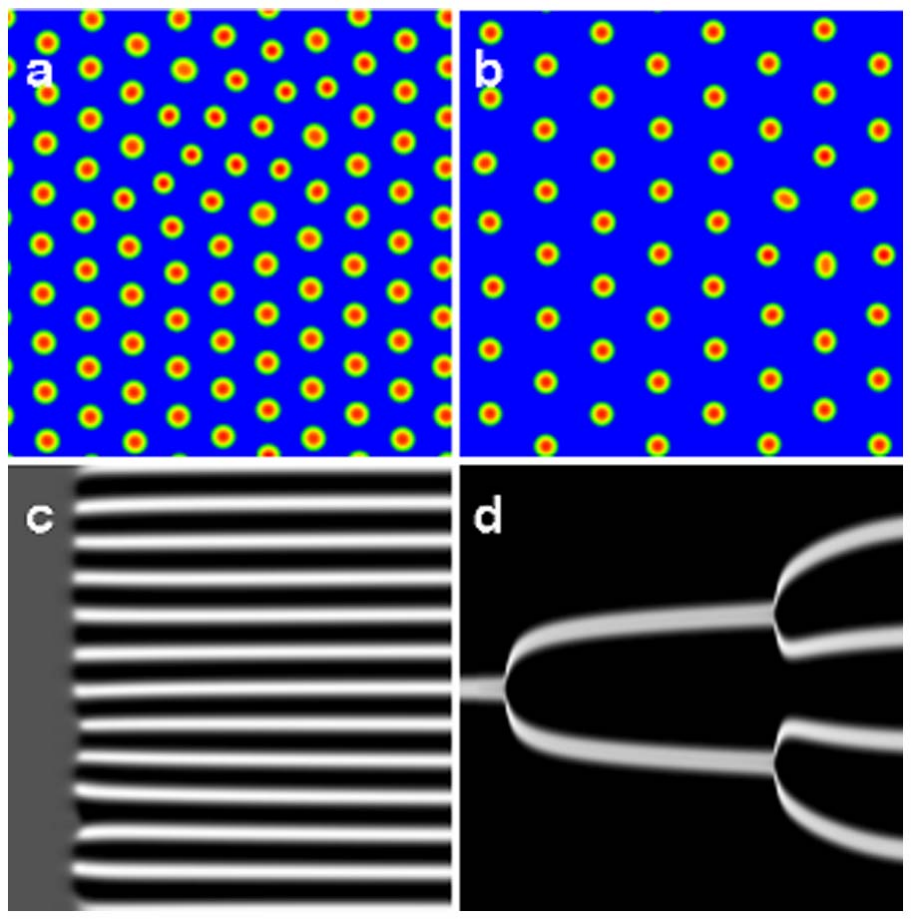
Stable stationary spot arrays in the reaction-diffusion system (1) generated by (a) Turing instability, (b) replication cascade. Two space dimensions are considered, with system size 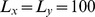 and periodic boundary conditions. Typical formation pathways for the Turing case (c) and the replication scenario (d) are shown in the space-time diagrams for simulations in one-dimensional space with 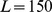. In (a–d), the variable 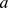 is displayed in color code: red, respectively white denote large values. Parameters: (a) 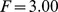; (b) 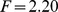; (c) 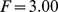, displayed time interval 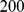, (d) 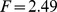, displayed time interval 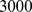. Other parameters as in Fig. 2. A pattern profile for both variables 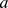 and 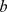 will be shown in Fig. 4(b).

While these examples show that similar phenomena appear in *different systems*, an even more intriguing feature is that patterns that look qualitatively similar can be generated by very *different mechanisms* in the *same system*. Consider the patterns shown in Figures 1(a,b), which are the result of numerical simulations of a typical bistable reaction-diffusion system in two spatial dimensions. While both figures represent stationary arrays of spots (increased concentrations of one or more chemical species relative to others), their evolutionary pathways are quite different. Figure 1(a) was generated by the Turing mechanism [15], i.e. from a uniform stationary state unstable under spatial perturbations, giving rise to a stationary, spatially periodic pattern. This is illustrated by a space-time diagram for a simulation in one space dimension in Fig. 1(c), where an initially uniform state almost simultaneously develops 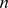 spots as a result of the small random perturbation.

In contrast to the above, the pattern in Fig. 1(b) was generated by perturbing a *different* uniform steady state, creating a single spot, that after a slight increase in the feed-rate, undergoes a replication cascade of spots, eventually filling the space (again illustrated in Fig. 1(d) by a space-time diagram for a simulation in one space dimension). Thus, while the asymptotic state of the system looks similar in both cases, the initial conditions, the parameter regimes in which they occur, and the mechanisms by which they are generated are different.

In the face of this, it is of interest to investigate if there is an abrupt transition or a smooth continuation –as a function of the feed-rate– between the patterns, as one traverses from one limit to the other. If there is a coexistence region, we want to investigate whether the asymptotic states of these patterns are identical and only the temporal evolution differ. Finally, since it may be desirable to select particular states of the system we seek to determine if it is possible to *use* the different mechanisms to smoothly engineer transitions between different states.

In this article, we explore these questions in a model reaction-diffusion system that displays both replication cascades *and* Turing instabilities. In the spirit of simplicity, tractability and clarity, we focus on a medium with only one spatial dimension and investigate the formation of patterns as a function of the feed-rate 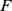. Therefore, we do not consider other pathways for the generation of spot solutions, such as transverse instabilities of stripe solutions (requiring at least two spatial dimensions). We find that, while the mechanisms driving the formation of spot arrays are discernibly separated in different regimes of 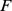, the patterns are essentially indistinguishable in intermediate regimes. Nevertheless, we find degeneracies, hysteresis and directionality effects that can be exploited for the purposes of pattern selection, via the tuning of the feed-rate.

## Results

### The model and basic instabilities

Our model reaction-diffusion system (first introduced in [16]) is described by the differential equations 
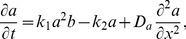
(1a)

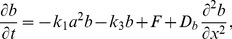
(1b)where 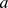 can be interpreted as the concentration of an activator 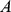 and 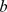 as the concentration of a substrate 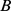. There is an autocatalytic step for 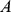 at rate 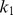, and decay reactions for 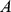 and 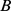 at rates 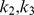, while 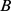 is fed in to the system at a rate 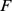. The model is closely related to a class of well-studied reaction-diffusion systems such as the Sel'kov-Gray-Scott model [17]–[19] (see also Sec. S1 of file S1 ), the Gierer-Meinhardt model [20] and the Brusselator [21].

We begin our analysis by first determining the uniform absorbing states of the model and then proceed to determine the specific instability associated with each state. Without loss of generality, the concentrations can be rescaled to 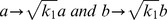; then the stationary uniform states are determined by setting the right-hand side of Eq. (1) to zero. Doing so, we obtain 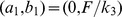, which we refer to as state **1**. At a critical value of the feed-rate 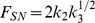, we find that two more solutions are generated by a saddle-node bifurcation. The first is an *unstable* intermediate state **2** and the second is a *stable* state **3** given by 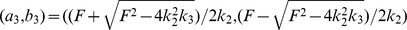. In addition to this we find that the system undergoes a Hopf bifurcation at yet another critical value 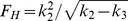, whereby in the range 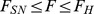, state **3** is potentially unstable with respect to temporal oscillations (for details see Sec. S2 of file S1).

Thus, the primary absorbing states of interest are **1** and **3**. These turn out to display distinct forms of instability. At a critical feed-rate 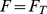, state **3** is linearly unstable with respect to spatially inhomogeneous perturbations, leading to the formation of Turing patterns in the interval 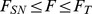 (see Sec. S3 of file S1). The characteristic wavelength of the pattern 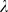 can be determined through a standard linear stability analysis, and this determines the total number of spots 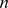 that are present in the system through the simple relation 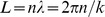, where 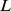 is the system size and 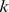 the wavenumber (see Methods).

On the other hand, while state **1** is stable with respect to infinitesimally small perturbations, it is *unstable* to localized large-amplitude perturbations, that can induce the formation of a single spot. Using the technique of scale-separation, one can calculate the profiles of the spot solutions, along with the parameter regimes for which they exist. In a particular limit, where 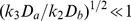, we can define a critical feed-rate for the formation of single-spot solutions, such that spots exist for 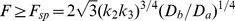 (details are shown in Sec. S4 of file S1). As 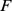 is further increased, the single spot becomes unstable with respect to a replication cascade (at a numerically determined critical feed-rate 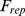) which eventually fills the system with a spot array, for related work for the Gray-Scott model see [22]–[30].

It is essential to point out that the fundamental difference between the formation of spot arrays via the Turing mechanism or via replication cascades, is that the former results from an instability of state **3** to infinitesimally small perturbations with a characteristic wavelength, while the latter is the result of a localized large-amplitude perturbation to state **1**.

### Turing patterns and localized spot patterns

We next investigate the differences between these two pattern formation mechanisms through the aid of numerical simulations, where we initialize the system in a variety of different initial states and examine the corresponding asymptotic states. To compare the generated patterns, we need to choose a suitable metric to distinguish them. In principle, there are many quantities one can measure, however, as Fig. 1 suggests, a particularly simple choice would be to simply count the number of spots 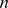 that are generated in the asymptotic state of the evolution of the system.

**Figure 2:**
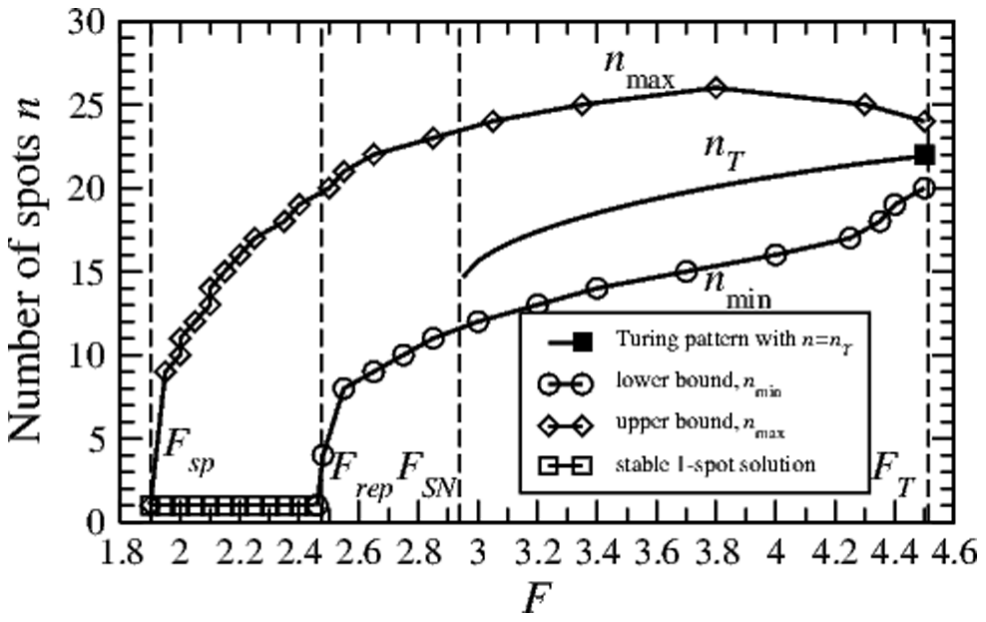
Stability area for 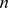-spot arrays as a function of 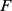 for a system size 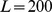 with periodic boundary conditions and 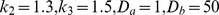 (details of simulation are covered in Methods). The stability area is enclosed by the curves 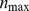 and 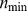, corresponding to the maximum and minimum number of stable spots for a given 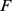. 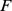 is changed in steps of 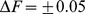 using the asymptotic state of the previous 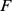 as initial condition (ramping). Turing patterns are marked by the curve 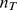. Vertical lines correspond to the values for the instabilities: 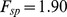, 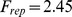, 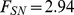, 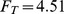.

Consider the plot in Fig. 2, where we show the number of spots 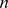 as a function of the feed-rate 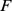 in the asymptotic state of the simulation (for the numerical details of the simulations, see Methods). We start with a single spot induced on the background of state **1** in the region 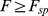 (that supports stable spots) and gradually increase 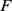 in small increments of 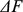. Doing so, we eventually reach a critical 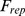 where the spot splits into two spots (replicates). The 2-spot solution may again be unstable, and the splitting process is repeated. This is the situation if we start with a *single* spot as initial condition. However, in the region 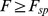, we can also directly create a 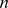-spot array with 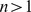 by inducing multiple large amplitude perturbations in different spatial locations of the system. The size of each spot of course is finite (being determined by the diffusion coefficients 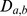) and consequently there is a maximum number of spots 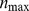 that can be supported in a finite medium. Thus in the region 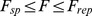 we can initialize a wide range of spot arrays within the bounds 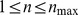 and by the same procedure of incrementing 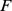, determine the values of 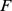 at which the spot array replicates. The resulting curve is displayed in Fig. 2 as the lower boundary of the stability area. These values of 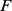 for each 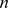 represent a generalization of the critical feed-rate 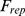 for 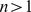. Clearly, this also implies that the curve corresponds to the *minimum* number of stable spots 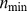 that can be supported by the system for fixed 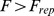, and we thus label this curve 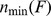.

We next turn our attention to the Turing regime (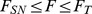) and the spot patterns found there. The onset of the Turing instability is of special interest: by inducing a small-amplitude perturbation around state **3** at 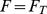, we obtain a *native* Turing pattern of 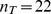 spots (denoted in Fig. 2 with a black square) in very good agreement with the theoretical value predicted by linear stability analysis (see Sec. S3 and Eq. (S9) of file S1). Away from 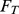, the analysis provides us with a continuous *band* of unstable wavelengths. Extensive simulations show that in the entire Turing regime (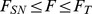), small random perturbations of state **3** lead *on average* to a spot pattern with 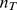 spots (marked by the solid curve extending from 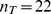 at 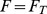 in Fig. 2), as predicted by linear stability analysis using the *most unstable* wavelength. This is in agreement with similar findings for the Gray-Scott model [31], confirming that patterns in this regime and initialized in this way are indeed bonafide Turing patterns.

Comparing the replication mechanism with the Turing mechanism, we recognize that the former provides an elegant way to access a number of spots that are *different* from 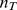 (the native Turing pattern) *within* the Turing regime. This is done by first initializing a 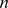-spot pattern for 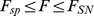 (outside the Turing regime), and then gradually increasing 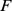 until we are within the Turing regime. In this way we can select a wide range of 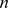 within the bounds 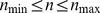 that differ from 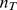. We note that Turing patterns with 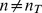 can also be generated by expanding fronts generated by perturbing state **1** in the Turing regime (see Sec. S5 of file S1), however this is not the focus of this article.

Furthermore, starting from any stable 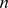-spot array, we are free to reverse the procedure and *decrease* 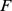 in increments of 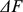. We find that after a particular value of 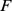 is reached, 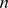 now *decreases*. By continuing this process and repeating it for all 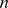, we obtain the upper curve in Fig. 2 that gives the *maximum* number of stable spots 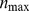 that can be sustained for a given 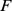. The area enclosed by the curves 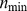 and 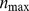 thus marks the stability region of 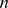-spot arrays as a function of the feed-rate 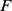. We immediately see from the figure that degenerate 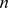-spot arrays exist for a large range of 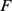, where the arrays can in principle be generated by *different* mechanisms.

Taken together, these results allow us to interpret 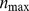 as a *disappearance* boundary where a 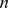 spot solution goes to a new value 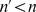, and 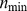 as a *splitting* boundary where 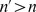. In general, in an infinite system, 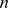 spots split into 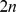 spots, however in a finite system this is constrained by its size. Therefore even in the region that supports replication, for large enough 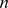, some of the spots in the array splits while other do not. The specific value of 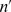 is sensitive to small perturbations, in particular at the moment the splitting or disappearance takes place.

**Figure 3:**
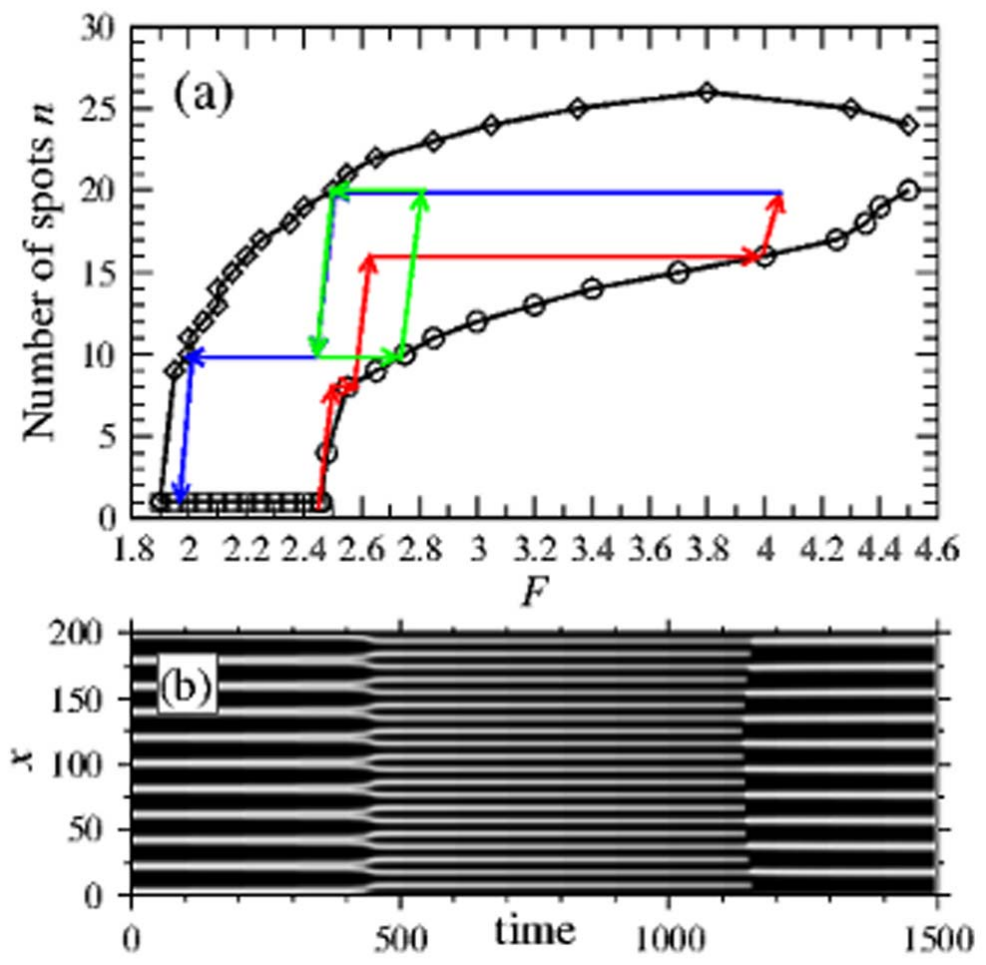
Different pattern pathways. (a) The red and blue pathways represent a hysteresis curve for an example 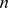-spot array induced in state **1**. We observe a sequence 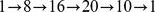 spots. The green path represents a cycle between 10 and 20 spots (more see text). (b) Space-time diagram for 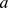 along the green path shown in (a). 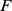 is changed about 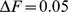 each 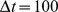. Simulation starts with a 10-spot solution at 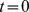 with 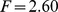, and 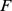 increases until 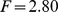, where splitting is observed. Then 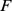 is decreased until 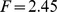, where 10 spots disappear, and after which it is increased again until 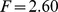 is reached.

Clearly, as we can create many different initial conditions, many different splitting or disappearance pathways exist. As an illustrative example we show one where a single spot is initialized on the background of state **1**. By increasing 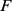, the solution reaches the boundary 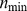 and splitting ocurrs. The resulting two spots are also unstable, and finally an 8-spot array is formed. By further increasing 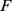, the array splits into 16 spots. Then it maintains stability for a wide range as 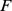 is increased further, well into the Turing regime, until it splits as it encounters 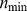 again. This evolution is shown via the red path in Fig. 3(a). If we now decrease 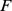, the boundary 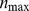 is encountered twice, and finally the number of spots decreases to 1 again (shown as a blue path in Fig. 3(a)). This is an example of a hysteresis curve connected to the degeneracy of the 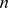-spot arrays.

Another example is shown by the green path in Fig. 3(a), where we cycle the spot-array solution between 10 and 20 spots. To illustrate how this cycle lookes in a real simulation, in Fig. 3(b) we show a space-time diagram for the variable 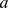 along the green path. We start from a 10-spot solution for 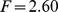, increase 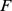 in small steps until splitting to a 20-spot solution is observed. Then, we decrease 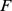 while preserving the 20-spot solution (hysteresis) until finally disappearance of spots takes place and the 10-spot solution is recovered.

The hysteresis effect clearly has the consequence of a preferred directionality in the system for inducing a replication pathway. Replication cascades proceed only via an *increase* in the feed-rate and for 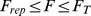. Conversely, the formation of a Turing pattern appears for a *fixed* 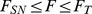 and for a particular class of initial conditions.

### Pattern profiles of 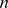-spot solutions

**Figure 4:**
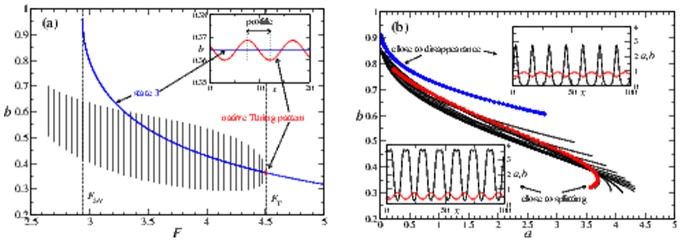
Pattern profiles. (a) The profile of 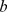 in a Turing pattern as a function of 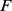 for fixed 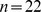. The blue curve represents the steady-state value 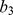. The vertical dashed lines 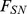 and 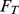 mark the Turing regime. A Turing pattern appears supercritically at 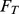 (inset) and its amplitude increases as one moves away from threshold. At a certain point, the profile ceases to oscillate around 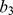, and continues to exist beyond the Turing regime without qualitative changes. (b) Multiple 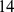-spot profiles in 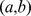 space, for 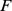 between 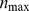 at 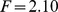 (blue curve) and 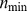 at 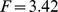 (red curve). The insets show the corresponding concentration profiles (black is 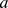, red is 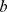).

While measuring 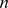 has been fruitful in determining the stability region of the solutions, it does not provide any detailed information about the spatial distribution of the pattern. Do the spot arrays created by Turing instability and spot arrays created by replications show any differences? Clearly, as one changes 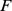 smoothly, the distribution of the concentration will vary, even as 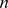 remains constant. A simple way of determining this is to measure the *profile* of the spots, which is the spatial range between its maximum and minimum concentrations. A visual illustration of this definition is provided in Fig. 4(a) inset.

To investigate this, we initialize a pattern with 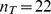 spots at the Turing boundary 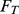 and examine the change in profile as we *decrease* 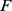. In Fig. 4(a), we plot the profile of 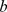 in function of 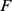. In the same figure we mark the existence region of state **3** by the dashed vertical boundary 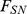 as well as the steady-state value 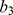 by a solid blue curve (note that state **3** exists only for 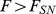). We find that close to 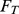 the amplitude of the pattern (marked by the vertical solid lines) is small, and the concentration of 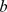 oscillates symmetrically around state **3**, in line with what is expected at 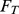 (see inset). However, as we move away from 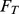, the amplitude increases and the profile shifts in phase space. At some point the pattern ceases to oscillate around state **3**, and eventually decouples from state **3**, continuing to persist even *below* 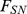. This decoupling occurs without any qualitative change as the pattern crosses the boundary. The implication of this is that for 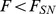 the persistent spot pattern can be interpreted as a continuation of a Turing pattern, although it is independent of state **3** (unlike a near-threshold Turing pattern) and no Turing analysis can be applied. In fact, spot arrays with same 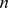, but created either through the Turing mechanism or replication cascades show no quantitative or qualitative difference, implying that arrays created by the two mechanisms are practically indistinguishable in the intermediate regime.

We next examine the change in profile as we vary 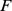 between the stability boundaries 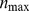 and 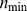, for an array with 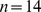 spots (note that for the 22-spot solution we do not reach the 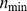 curve). In Fig. 4(b) we represent the resulting patterns in the space of the concentrations 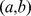. Again we see that the patterns change continuously as 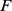 is varied, from the blue curve for 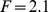 to the red curve for 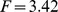. For the former, a spatial plot of the pattern (inset upper right) reveals that it is sharply peaked and that a spot has a small extension. If one perturbs the system by further decreasing 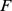 by a small amount, the number of spots decreases. Turning our attention to the other boundary 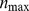, an examination of the profiles there reveals the existence of degenerate values of 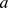 for fixed 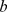 (marked in red). This implies that within the spot, a small dip in the center is formed, as visualized in the inset (lower left). Now, as one increases 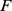 by a small amount, the spot pattern eventually splits along this dip.

## Discussion

In conclusion, using a simple reaction-diffusion model, we have identified the stability region for 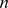-spot solutions in the parameter space spanned by a natural control parameter (the feed-rate 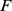). In general, for a given 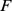, we find multistability of spot solutions, with a range of spot numbers 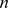, bounded by numerically determined curves 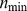 and 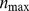.

Spot arrays in the reaction-diffusion system (1) can be created in very different ways, with two distinct limiting behaviors (single-spot solution and native Turing pattern). These arrays are indistinguishable in intermediate regimes (the asymptotic states for fixed 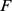 and 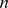 are identical) where both generative mechanisms coexist. This means that either mechanism can be used to generate the same pattern. Therefore, to discriminate between the pattern formation mechanisms is to some degree artificial, as these can only be distinguished during their transient phases. However, due to the different transients in each case, the initial conditions determine the pattern evolution and the final number of spots in a non-trivial manner: While small random perturbations create typical Turing patterns with 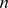 coinciding on average with 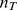, through an appropriate tuning of 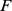, we gain access to a wider range of 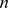 via replication cascades. As we have shown, one can make use of the hysteresis feature of the system to generate periodic cycles of spot replication and destruction.

Despite the simple and specific chemical nature of our model, we expect the qualitative result to hold for similar non-chemical systems and in general for those complex scenarios whose dynamics (possibly in reduced form) can by described by reaction-diffusion models such as certain fluid systems [11]–[14]. There, cycles of spot replication and destruction could be used to engineer transitions between out-of-equilibrium states. For example, splitting of turbulent stripes is dominant for large Reynolds numbers in plane Couette flow, while for low Reynolds numbers stripe decay is favored [12]. While the specifics in that system are different from our model, analogous to the role played by the feed-rate, we hypothesize that it could be possible to control the number of stripes through switching of the Reynolds number.

As perspectives for future work we mention the possibility to engineer the system by modulating the feed-rate in time, using a self-generated signal (*feedback*) that can use the splitting/disappearance pathways [32]. Furthermore, transitions between spot arrays with different 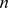 can be also induced by application of noise. However, the realization of these ideas goes beyond the scope of this article.

In the spirit of simplicity, tractability and clarity, we have focused on a medium with one spatial dimension. Obviously, the dynamics of localized spots and Turing patterns is much richer in two space dimensions. However, we expect that that the main result of this study holds qualitatively also for two-dimensional spot arrays.

## Methods

The numerical simulations of Eq. (1) were conducted in a one-dimensional space of size 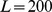 with periodic boundary conditions which ensures that there no spots attached to the boundary (varying 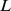 as well as using no-flux boundary conditions have not shown to produce mayor changes). A spatial grid with 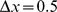 was used along with a Euler routine for time integration and a 3-point stencil for the diffusion operator. In order for increased accuracy for patterns close to instabilities and for validation purposes, a 4th-order Runge-Kutta scheme was employed along with a smaller grid resolution 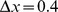 and a 5-point stencil. The two-dimensional simulations shown in Fig. 1(a,b) are only for the purpose of illustration; they correspond to simulations with 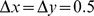 and a 5-point stencil for the diffusion operator.

We are not interested in oscillatory behavior and therefore choose 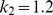 and 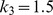 in order to be far from the Hopf bifurcation curve (compare Fig. S1 of file S1). In order to observe localized spot and Turing patterns, sufficiently strong substrate diffusion is necessary, and we set 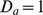 and 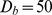 accordingly. Although for one-dimensional localized patterns, the notation *spike* is used in the literature, we apply the more general notation *spots*.

To obtain the limiting curves in Fig. 2, spot solutions are initialized for different 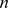 in the region 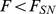. The asymptotic state of a simulation is determined at 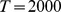, although transients usually have died out after 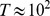 (if 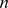 changes within the simulation) or 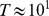 (if 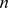 does not change within the simulation). Following this, 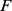 is increased in increments of 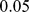, and the simulation is allowed to run again until the asymptotic state is reached. This procedure is repeated until splitting is observed. In the same way, 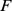 can be either increased further or decreased until spots split again or disappear. This iterative process has been exhaustively performed for all possible 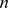 to determine the stability area.

We note that the numerical results come with inherent imprecisions, in particular for large 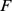 where the amplitude of the Turing pattern vanishes and for small 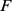 where the spot pattern disappears. Finite simulation time may mistake a transient for an asymptotic state. Also, the finite size of the medium (together with the periodic boundary conditions) implies that the range of 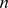 (which is a positive integer number) is limited. However, simulations for larger system size and no-flux boundary conditions have not revealed qualitatively new behavior, though of course 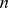 increases and the curves in Fig. 2 are extensive in system size.

## Supporting Information

File S1 (PDF)
