# Supplementary material for: Parametric Pattern Selection in a Reaction-Diffusion Model
Source: PLoS One. 2013 Oct 29;8(10):e77337. doi: 10.1371/journal.pone.0077337 (PMC3812206; doi:10.1371/journal.pone.0077337)
Supplement: File S1 — (PDF) [file pone.0077337.s001.pdf]

Supplementary Material

Parametric pattern selection in a reaction-diffusion model
Michael Stich, Gourab Ghoshal, and Juan Pérez-Mercader

Table of Contents

S1 Connection with the Gray-Scott model **S-2**

S2 Hopf analysis **S-2**

S3 Turing analysis **S-4**

S4 Spot analysis **S-5**
S4.1 Example solution S-6

S5 Expanding fronts **S-7**

References **S-8**

List of Figures

S1 Phase diagram in (F, k_3) space. S-3
S2 Space-time plots for the formation of spot arrays. S-8

S1 Connection with the Gray-Scott model

If we consider Eq. (1) without diffusion, we have

$$\frac{da}{dt} = k_1 a^2 b - k_2 a, \tag{S1a}$$
$$\frac{db}{dt} = -k_1 a^2 b - k_3 b + F. \tag{S1b}$$

By rescaling $a \to (k_3/F)a$, $b \to (k_3/F)b$, $t \to (k_1 F^2/k_3^2)t$, we obtain a rescaled Gray-Scott model of the form introduced by Pearson [1].

$$\frac{da}{dt} = a^2 b - (F_{GS} + k_{GS})a, \tag{S2a}$$
$$\frac{db}{dt} = -a^2 b + F_{GS}(1 - b), \tag{S2b}$$

where

$$F_{GS} = \frac{k_3^3}{k_1 F^2}, \tag{S3a}$$
$$k_{GS} = \frac{k_3^2}{k_1 F^2}(k_2 - k_3). \tag{S3b}$$

and GS refers to Gray-Scott. It is apparent that k_{GS} takes on unphysical negative values for $k_3 > k_2$, and thus a one-to-one correspondence between the two models is strictly valid for $k_2 \geq k_3$.

S2 Hopf analysis

To determine the stability of the steady state solutions of Eq. (1) we perform a linear stability analysis and examine the eigenvalues of the Jacobian matrix which are:

$$\lambda_{1,2} = -\tfrac{1}{2}\left(\alpha \pm \sqrt{\alpha^2 - 4\theta}\right), \tag{S4}$$

where

$$\alpha = k_2 + k_3 + a^2 - 2ab, \tag{S5a}$$
$$\theta = k_2 k_3 + k_2 a^2 - 2k_3 ab, \tag{S5b}$$

and the concentrations (a, b) are understood to denote their steady-state values. For stability we require $\lambda_i < 0 \ \forall \ i$. It is straightforward to show that for any realistic choices of parameters and concentration values, state 1 is always stable while the intermediate state is always unstable. We thus focus our attention on state 3.

Onset of instability occurs when the eigenvalues cross zero in the imaginary axis. From Eq. (S5), we see that this is satisfied when $\alpha = 0$ provided $\theta > 0$ which is necessarily true for $F \geq F_{SN}$ (the region of existence for state 3) resulting in a pair of complex conjugate eigenvalues $\pm\sqrt{-\theta}$. This is

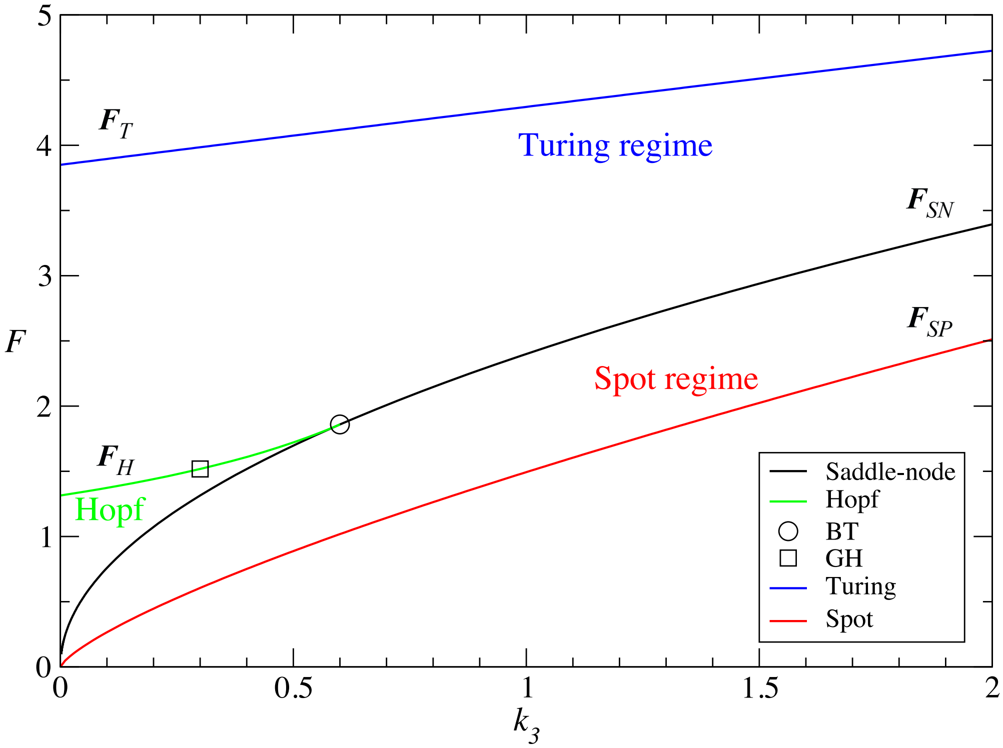

Figure S1: Phase diagram for the system governed by Eq. (1) in the (F, k_3) space, ($k_2 = 1.2, D_a = 1, D_b = 50$). The state 1 exists in all parameter regions, while state 3 exists above F_{SN} (black curve). Below F_T (blue curve), state 3 is Turing-unstable. Existence of single spot solutions (as large amplitude perturbations to state 1) are possible for $F > F_{sp}$ as marked by the red curve. The region enclosed by F_H (green curve) and F_{SN} permit temporal oscillations.

a sign of a Hopf bifurcation which occurs at the curve,

$$F_H = \frac{k_2^2}{\sqrt{k_2 - k_3}}. \tag{S6}$$

Above this curve state 3 is stable whereas below it may display oscillations. For $k_3 = k_2/2$, $F_{SN} = F_H$ and the saddle-node curve collides with the Hopf curve at a Bogdanov-Takens (BT) point beyond which the Hopf curve ceases to exist. Thus for $k_3 > k_2/2$ state 3 is always stable and does not display any oscillations. For $k_3 < k_2/2$, below the Hopf curve there may be stable oscillations depending on whether the bifurcation is subcritical or supercritical. In the subcritical case state 3 undergoes a bifurcation from a stable focus surrounded by an unstable limit cycle to an unstable focus (and only then state 1 remains as an attractor). In the supercritical case, a stable focus converts into an unstable focus surrounded by a stable limit cycle such that the concentrations may display stable oscillations. The point separating the super- and subcritical bifurcations is referred to as a Generalized Hopf (GH) point, located at $k_3 = k_2/4$. The Hopf curve along with the BT and GH points are shown in Fig. S1.

S3 Turing analysis

To determine the conditions for the Turing instability, we employ the ansatz

$$c_i = c_i^0 + c_i' \mathrm{e}^{\lambda t} \mathrm{e}^{\mathrm{i}qx}, \tag{S7}$$

for $i = 1, 2$ (corresponding to a, b) where λ is the eigenvalue and q a wavenumber. Inserting Eq. (S7) into Eq. (1) we obtain an equation identical to Eq. (S4), however, now with

$$\alpha = k_2 + k_3 + (D_a + D_b)q^2 + A_1^2 - 2ab, \tag{S8a}$$

$$\theta = (k_2 + D_a q^2)(k_3 + D_b q^2) + (k_2 + D_a q^2)a^2 - 2(k_3 + D_b q^2)ab. \tag{S8b}$$

For the equilibrium solutions to be unstable, we require that the real part of the eigenvalues change sign from negative to positive. It is straightforward to show that $\alpha > 0$ for any non-zero positive value for q, therefore we look for a non-zero positive value for q that makes $\theta = 0$ such that $\lambda = 0$ (cf. Eq. (S4)). State 1 is always stable for any q value so we focus on state 3.

We notice that θ is a parabola in the quantity q^2 that opens upwards, being positive for $q^2 = 0$ (for temporally stable solutions) and positive for large q^2. A condition for linear instability in the presence of diffusion is then obtained by determining the point at which the minimum of the parabola first becomes negative. Setting the derivative of θ with respect to q^2 to zero, we learn that the minimum occurs at the wavenumber q_c given by

$$q_c = \left[\frac{2D_b k_2^3 - D_a F \left(F + \sqrt{F^2 - 4k_2^2 k_3} \right)}{4 D_a D_b k_2^2} \right]^{1/2}. \tag{S9}$$

We also require that this wavenumber be real (guaranteed by $F > F_{SN}$) and positive, leading to the condition

$$F > \frac{\sigma k_2^2}{\sqrt{\sigma k_2 - k_3}} \tag{S10}$$

where $\sigma = D_b/D_a$. Substituting q_c into θ and setting it to zero then gives the Turing curve:

$$F_T = \left[\frac{\sigma k_2^3 (3\sigma^2 k_2^2 + 7\sigma k_2 k_3 + 8k_3^2) - 2\sqrt{2}\sqrt{\sigma^3 k_2^7 (\sigma k_2 - k_3)(\sigma k_2 + k_3)^2}}{(\sigma k_2 + k_3)^2} \right]. \tag{S11}$$

The positivity of q_c together with (S10), enforces the inequality

$$k_3 < \sigma \frac{k_2}{2}. \tag{S12}$$

At this point $F_T = F_{SN}$ and the Turing curve collides with the saddle-node curve, beyond which it ceases to exist. For $\sigma = 1$ this happens at the BT point, while σ can be adjusted, such that there is overlap (or lack thereof) with the Hopf regime. F_T is shown as a blue curve in Fig. S1. For the parameter set $k_2 = 1.3, k_3 = 1.5, D_a = 1, D_b = 50$, we find $F_T = 4.5116$ and $q_c = 0.6897$. Therefore, a Turing pattern with wavenumber q_c corresponds to a n-spot pattern with $n = Lq_c/2\pi$ spots in a system of size L, in our case ($L = 200$) to a spot number of $n = 21.9544$.

While at threshold, there is an unique unstable mode with wavenumber q_c, above threshold, there is whole band of unstable wavenumbers, i.e., modes with a positive growth rate (real part of the eigenvalue λ of the linear stability analysis). The most unstable mode q_{max} can be numerically determined through the condition

$$\frac{d}{dq} Re(\lambda) = 0. \tag{S13}$$

The resulting q_{max} can be transformed into a number of spots by $n_T = Lq_{max}/2\pi$, where L is the size of the medium.

It is important to point out that not every unstable wavenumber q (or n) corresponds to a stable pattern. A weakly-nonlinear analysis can determine the set of wavenumbers that give rise to *stable* n-spot patterns.

S4 Spot analysis

In our spot analysis, we follow the work done by Muratov and Osipov [2, 3], who—starting from the Gray-Scott model—have extensively studied pattern formation in reaction-diffusion systems of the form:

$$\begin{aligned} \tau_a \dot{a} &= l_a^2 \nabla^2 a - q(a, b, \theta), \\ \tau_b \dot{b} &= l_b^2 \nabla^2 b - Q(a, b, \theta), \end{aligned} \tag{S14}$$

where a is the activator and b is the inhibitor/substrate. The functions q, Q are the interaction terms, θ is a constant parameter (such as the feed-rate F), $\tau_{a_{1,2}}$ are their respective time-scales and $l_{a_{1,2}}$ are their characteristic length scales of variation. They show that in general, for localized pattern to emerge, the limit $l_a \ll l_b$ must be satisfied. This is interpreted as the length scale of the activator a varying on a much smaller scale than the inhibitor/substrate b. The formation of spikes/spots occurs in the background of the uniform state 1 and when it does, the size of the spot is of $O(l_a)$. Furthermore the stability of this pattern to perturbations is determined by the relative time scale of variation τ_a/τ_b. Therefore, the spot is characterized by a sharp peak of the activator, its variation in space must be much smaller than the inhibitor. When its decay rate is smaller than that of the inhibitor, it will be stable. However when it decays faster than the inhibitor (or indeed on the same order) then various kinds of instabilities may manifest themselves, among them spot replication.

To compare our system with the general form (S14), we first rescale Eq. (1) via the following transformations:

$$a = \frac{a}{k_2 \alpha}, \tag{S15a}$$

$$b = \frac{b}{F/k_3}, \tag{S15b}$$

where

$$\alpha = \left(\frac{k_3}{k_1}\right)^{1/2}. \tag{S16}$$

Note that the steady-state value of b in state 1 is F/k_3, thus the rescaling of the equations is with

respect to deviations from state 1. After rescaling we get

$$\tau_a \dot{a} = l_a^2 \nabla^2 a - (a - \frac{F}{k_2 \alpha} a^2 b), \tag{S17a}$$

$$\tau_b \dot{b} = l_b^2 \nabla^2 b - (b + a^2 b - 1). \tag{S17b}$$

The characteristic time scale for each species are:

$$\tau_a = k_2^{-1}, \quad \tau_b = k_3^{-1}.$$

We also notice a characteristic length scale for the species $a_{1,2}$ defined as

$$l_a = \sqrt{D_a \tau_a}, \quad l_b = \sqrt{D_b \tau_b},$$

which can now be interpreted as a diffusion length. Focusing our attention on a, b and comparing (S14) with (S17) we see that:

$$q(a, b, \theta) = a - \frac{F}{k_2 \alpha} a^2 b, \quad Q(a, b, \theta) = b + a^2 b - 1, \quad \theta = \frac{F}{k_2 \alpha}.$$

We find it convenient to define the following dimensionless parameters:

$$\begin{aligned} \tilde{\tau} &= \frac{\tau_a}{\tau_b} \equiv \frac{k_3}{k_2}, \\ \tilde{l} &= \frac{l_a}{l_b} \equiv \sqrt{\frac{\tilde{\tau}}{\sigma}}, \end{aligned} \tag{S18}$$

where $\sigma = D_b / D_a$. In the limit $\tilde{l} \ll 1$, we can re-write the equations for a, b as,

$$\begin{aligned} \tilde{\tau} \dot{a} &= \tilde{l}^{\,2} \nabla^2 a + \theta a^2 b - a, \\ \dot{b} &= \nabla^2 b + 1 - b^2 a - b. \end{aligned} \tag{S19}$$

S4.1 Example solution

In general, different types of spike solutions (in terms of its profile) will exist in the different regimes of the limit $\tilde{l} \ll 1$. As an example, we provide a simple case which is accurate in the limit $\tilde{l} \simeq \theta^2 \ll 1$. In one dimension and in the steady-state Eq. (S19) reduces to

$$\tilde{l}^{\,2} \frac{d^2 a}{dx^2} + \theta a^2 b - a = 0, \tag{S20}$$

$$\frac{d^2 b}{dx^2} + 1 - b^2 a - b = 0. \tag{S21}$$

Since b varies on the order of unity, and a varies on the order of $\tilde{l} \ll 1$, one can separate scales *inside* and *outside* the spike. Assuming that within the spike $b = b_s$ (s denotes spike) is roughly a constant, substituting it into the first equation in (S21) and solving for a we get,

$$a(x) = a(0) \cosh^{-2} \left(\frac{x}{2\tilde{l}} \right) \text{ with } a(0) = \frac{3}{2\theta b_s},$$

where $a(0)$ is the amplitude of the spike. Away from the spike, $a = 0$ (since this is in the background of state 1). Substituting this into the second equation in (S21) and solving for b we have

$$b(x) = 1 - \frac{3}{b_s}\frac{\tilde{l}}{\theta^2}\mathrm{e}^{-|x|}.$$

Matching this solution with the condition $b(0) = b_s$ we get the following expressions:

$$a(0) = \frac{3\theta}{\theta_c}\left[1 \pm \sqrt{1 - \left(\frac{\theta_c}{\theta}\right)^2}\right], \quad b_s = \frac{1}{2}\left(\frac{\theta_c}{\theta}\right)^2\left[1 \pm \sqrt{1 - \left(\frac{\theta_c}{\theta}\right)^2}\right]^{-1},$$

where $\theta_c = \sqrt{12\tilde{l}}$. We see thus that if $\theta < \theta_c$, the spike solution does not exist, whereas for $\theta \geq \theta_c$ there are two solutions. The one corresponding to the positive sign has larger amplitude and is always stable. Whereas the lower amplitude solution is always unstable.

Along similar lines for the critical feed rates $F_{SN,H,T}$ (corresponding to the saddle-node, Hopf and Turing bifurcations in the previous section) we can define a critical feed rate F_{sp} for the formation of spots. Using the condition $\theta \geq \theta_c$ we find,

$$F_{sp} = 2\sqrt{3}(k_2 k_3)^{3/4}\left(\frac{1}{\sigma}\right)^{1/4}, \tag{S22}$$

such that spots exist for $F \geq F_{sp}$. In Fig. S1 we plot F_{sp} as a red curve for qualitative purposes, keeping in mind that this is an approximate solution and breaks down with increasing k_3.

S5 Expanding fronts

An alternative mechanism leading to the formation of Turing patterns is provided by expanding fronts. In particular, a perturbation of state 1 within the Turing regime, can lead to an expanding front that in its wake leaves a stationary periodic spot pattern (similar to what has been observed in the Gray-Scott model [4]). In Fig. S2, we show three examples of this for a fixed $F = 2.96$.

In the left panel, we see the result of perturbing state 1, where a slowly expanding front leaves a spot array in its wake. In the central panel a small *localized* perturbation to state 3 leads to a much faster expanding front than in the previous case. This is because state 3 is unstable in the Turing regime, while state 1 is stable. In the right panel we show the result of a slightly different perturbation to state 3 leading to an array with *different* n than in the middle panel.

The figures thus illustrate that the asymptotic state of patterns generated by this mechanism is quite sensitive to initial conditions. An averaging over an exhaustive state of initial conditions is thus required to determine the statistical properties of the dynamics. However, this is beyond the scope of this paper.

References

[1] Pearson JE (1993) Complex patterns in a simple system. Science 261: 189–192.

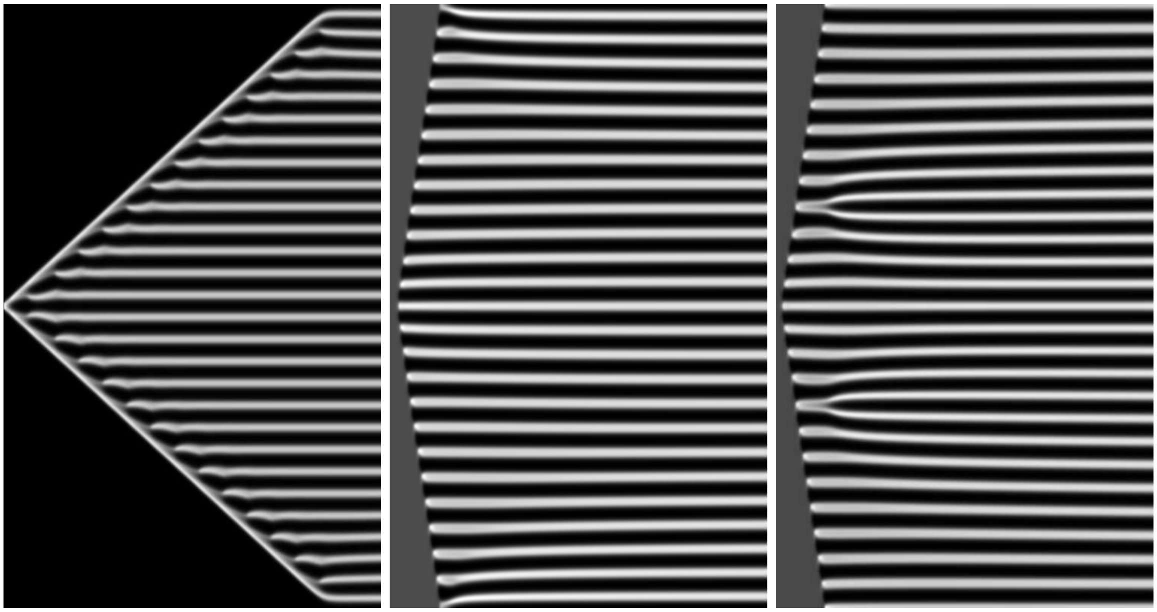

Figure S2: Space-time plots for the formation of spot arrays by expanding wavefronts. (Left) Spot array formed from a large-scale perturbation of state 1, 28 spots are formed. (Center) Spot array formed from a small perturbation of state 3, 25 spots are formed. (Right) Spot array formed from a different, but also small perturbation of state 3, 26 spots are formed. System size $L = 400$, time interval $t = 500$, $F = 2.96$.

[2] Muratov CB, Osipov VV (2000) Static spike autosolitons in the Gray-Scott model. J Phys A Math Gen 33: 8893–8916.

[3] Muratov CB, Osipov VV (2002) Stability of the static spike autosolitons in the Gray-Scott model. SIAM J Appl Math 62: 1463–1487.

[4] Mazin W, Rasmussen KE, Mosekilde E, Borckmans P, Dewel G (1996) Pattern formation in the bistable Gray-Scott model. Math Comput Simul 40: 371–396.
